# Integrative Analysis of Immunological Data to Explore Chronic Immune T-Cell Activation in Successfully Treated HIV Patients

**DOI:** 10.1371/journal.pone.0169164

**Published:** 2017-01-03

**Authors:** Marie-Quitterie Picat, Isabelle Pellegrin, Juliette Bitard, Linda Wittkop, Cécile Proust-Lima, Benoît Liquet, Jean-François Moreau, Fabrice Bonnet, Patrick Blanco, Rodolphe Thiébaut, ANRS CO3 Aquitaine Cohort

**Affiliations:** 1 Centre INSERM U1219 Bordeaux Population Health, Bordeaux, France; 2 Univ. Bordeaux, ISPED, Bordeaux, France; 3 Service d’Information Médicale, USMR, CHU de Bordeaux - Pôle de Santé Publique, Bordeaux, France; 4 INRIA, Team SISTM, Bordeaux, France; 5 Vaccine Research Institute-VRI, Hôpital Henri Mondor, Créteil, France; 6 Laboratoire d’Immunologie-Immunogénétique, Pôle de Biologie, CHU Bordeaux, Bordeaux, France; 7 Laboratoire de Mathématiques et de leurs Applications, Université de Pau et des Pays de l’Adour, UMR CNRS 5142, Pau, France; 8 ARC Centre of Excellence for Mathematical and Statistical Frontiers, Queensland University of Technology (QUT), Brisbane, Australia; 9 Univ. Bordeaux, Bordeaux, France; 10 CNRS, UMR 5164, Bordeaux, France; 11 Service de Médecine Interne et maladies Infectieuses, CHU de Bordeaux, Bordeaux, France; University of Cape Town, SOUTH AFRICA

## Abstract

**Objectives:**

To unravel the complex relationships between cytomegalovirus-induced-, autoimmune-induced responses, microbial translocation and chronic immune activation (CIA) in successfully treated HIV-infected patients and to explore the mediating role of alpha-interferon in these processes.

**Design:**

Cross-sectional study nested in the ANRS CO3 Aquitaine Cohort, a prospective hospital-based cohort of HIV-1-infected patients in South-Western France.

**Methods:**

Patients initiated antiretroviral therapy between 2005 and 2008 and were treated with sustained virological suppression for at least two years. CIA was defined by the percentage of HLA-DR+/CD38+ among CD8+T-cells. Integrative analyses were performed using structural equation modelling (SEM).

**Results:**

The main analysis was performed in 57 HLA-A*0201 positive patients, due to availability of percentages of actin-, vimentin-, lamin-specific CD8+T-cells (HLA-A2-restricted tests) to further characterize autoimmune response. Cytomegalovirus-induced response was assessed by Quantiferon and pp-65 ELISPOT. SEM revealed a direct effect of cytomegalovirus-induced response on CIA (standardized estimate βstd = 0.56, p-value = 0.0004). The effect of autoimmune-induced response on CIA was indirect through alpha-interferon pathway, assessed by expression levels of 5 alpha-interferon-stimulated genes ADAR, ISG15, IFIT1, Mx1 and OAS1 (effect of autoimmune response on alpha-interferon: βstd = 0.36, p-value = 0.0401; effect of alpha-interferon on CIA: βstd = 0.39, p-value = 0.0044). There was no direct effect of autoimmune-induced response on CIA (p-value = 0.3169). Microbial translocation as measured by 16SrDNA and sCD14 in plasma was not associated with CIA. Results were consistent in 142 patients in whom cytomegalovirus and auto-immunity responses were measured by Quantiferon and anti-nuclear antibodies, respectively. All analyses performed in HLA-A*0201 positive patients and in the overall population revealed a significant effect of IFN-α latent variable on CIA.

**Conclusion:**

The role of cytomegalovirus-induced response on CIA was confirmed as well as the involvement of alpha-interferon on CIA. The indirect effect of auto-immunity response on CIA revealed through the alpha-interferon pathway requires further investigation to confirm the potential role of auto-immunity for CIA in HIV-infected patients.

## Introduction

Immune activation is a leading factor for human immunodeficiency virus (HIV) disease progression [1, 2]. The persistence of immune activation is associated with both acquired immunodeficiency syndrome (AIDS) and non-AIDS comorbidities [3–6] in patients under successful antiretroviral therapy with long-term virological suppression. Noteworthy, chronic type I alpha-interferon (IFN-α) is a hallmark in chronic activation and has been reported as a factor influencing disease progression in persistent infections [7–9], including HIV [10–12]. The mechanisms driving chronic immune activation and type I interferon secretion are multifactorial and are not completely understood [13]. Cytomegalovirus (CMV) and microbial translocation have been proposed as triggering factors for persistent immune activationassociated with HIV-1 infection [14]. Microbial translocation and associated biomarkers, e.g. sCD14 and circulating plasma lipopolysaccharide [15–17], have been found to be implicated in the generation and maintenance of chronic immune activation. CMV is highly prevalent in HIV patients and may reactivate more frequently [18]. Indeed, HIV disrupts the balance between the host and coinfecting or dormant microbes, worsening control of these potential pathogens. HIV-infected adults are more likely to have subclinical bursts of CMV replication compared to the general population. CMV reactivation and replication begin due to a progressive loss of immune function, and, in particular, due to a loss of cell-mediated immunity. Increased CMV-specific antibodies and/or T cells have been associated with atherosclerosis [19, 20] and impaired CD4 T-cell reconstitution [18] in HIV-infected patients on cART. Altogether, these data suggest that CMV coinfection may be a driver of persistent immune activation. Indeed, a randomized clinical trial with valgancyclovir in CMV-seropositive cART-treated patients (n = 30) found that both CMV DNA and expression of CD38+HLA DR+ on T cells declined significantly in patients given valgancyclovir therapy [21]. In parallel, auto-immune induced immune activation has also been suspected as a driver for immune activation but an association is less consistently found [22]. Hence, an increase in the proportion of autoreactive T-cells has been shown by Rawson et al [23].

The contribution of each of these factors potentially varying from patient to patient and over the course of the disease is not well described. Using univariable and multivariable linear regressions, we previously evaluated the impact of cytomegalovirus-induced, microbial translocation and autoimmune-induced immune responses on chronic immune activation in 191 HIV-1-infected patients with long-term virological suppression while on antiretroviral therapy combination in the ACTHIV substudy of the ANRS CO3 Aquitaine cohort [24] (S1 Table for summary results). We reported that the immune response against CMV was independently associated with a higher percentage of CD8+ T cell activation as measured by HLA-DR+/CD38+CD8+T-cells. In contrast, autoimmune response and microbial translocation were not associated with chronic immune activation in adjusted analyses. However, two difficulties limited these analyses. Firstly, each process can be measured by several biomarkers. For instance, the immune response to CMV can be measured by Quantiferon-CMV-, CMV-pp65-ELISPOT- and CMV-pp65-specific-CD8+T-cell-positivity. Therefore, each multivariable linear regression analysis considering only one marker does not reflect the whole process. Secondly, the mechanism leading to chronic immune activation is complex, multifactorial and involved intermediate pathways such as type I interferon. Autoimmunity, autoimmune diseases and the type I interferon system are closely linked. For example, a type I interferon signature can be found in many autoimmune disorders including lupus, dermatomyositis or type I diabetes. The mechanisms involved in type I interferon secretion in patients implicate the immune complexes-mediated activation of endosomal TLR activation by self DNA [25, 26]. In chronic HIV patients it has been shown that the cross-presentation of caspase-cleaved self-antigen by dendritic cells was promoting the expansion and activation of self-reactive CD8+ T cells against vimentin [23]. A similar mechanism has been described in systemic lupus erythematosus patients in whom type I interferon promotes the differentiation of monocytes into dendritic cells, which are able to engulf apoptotic bodies and to present autoantigens to the adaptive immune system. Conversely, vimentin is an autoantigen targeted by the immune response in systemic lupus erythematosus patients [27]. In parallel, cytomegalovirus infection may also be able to stimulate type I IFN-α production through interferon-stimulated-genes [28].

We investigated the hypotheses of direct effects of CMV-induced, autoimmune-induced immune responses measured by percentages of actin-, vimentin-, lamin-specific CD8+T-cells and microbial translocation on chronic immune activation as well as indirect effects mediated by IFN-α gene expression, in successfully treated HIV-infected patients. We aimed at unravelling the complex relationships between CMV-induced-, autoimmune-induced immune responses, microbial translocation and chronic immune activation in these patients and to explore the mediating role of the IFN-α in these processes. We carried out an entirely new work including new data from gene expression analysis which were not presented in the former study. Furthermore we realized a distinct and original approach by using an integrative structural equation modelling analysis [29].

## Methods

### Population

The ACTHIV study [24] is a cross-sectional study nested in the ANRS CO3 Aquitaine Cohort [30], a prospective hospital-based cohort of HIV-1-infected patients, in South-Western France. Patients initiated antiretroviral therapy between 2005 and 2008, and were treated with sustained virological suppression (HIV-1 RNA load below the detection limit of 50 copies/mL) for at least two years. Exclusion criteria were two consecutive HIV-1 RNA above 50 copies/mL, hepatitis C virus or hepatitis B virus coinfection, and signs of acute infection. The ACTHIV protocol was approved by the Bordeaux University Institutional Review Board. All patients signed an informed consent before participating in the study.

### Assessments of immune responses

Available biomarkers in the ACTHIV study for assessing CMV-induced-, autoimmune-induced immune responses, microbial translocation, quantitation of IFN-α gene expression and chronic immune activation are presented below. Methods for laboratory measurements assessed by flow cytometry have been reported in Wittkop et al [24] (Material and Methods subsection entitled “Laboratory measurements”).

#### Cytomegalovirus-induced immune response

The CMV-induced immune response was determined by CMV seropositivity and using Quantiferon CMV analysis (Cellestis, Australia), CMV-pp65 enzyme-linked immunosorbent spot (EliSpot) analysis, and HLA-A0201 CMV-pp65-specific tetramers CD8+. CMV seropositivity was assessed by detecting immunoglobulin G and immunoglobulin M antibodies to CMV late antigens (Dade Behring, Deerfield, IL). Quantiferon is a trademark of the Cellestis company, now sold by Quiagen. QuantiFERON-CMV uses three specific blood collection tubes that are coated with peptides simulating CD8+-specific epitopes of CMV proteins, along with negative and positive control tubes. Stimulation of CD8+ T-cells in 1 ml of whole blood with the CMV peptides induces in the production of IFN-γ in infected individuals. An ELISA is then used to measure the amount of IFN-γ present in plasma from each of the 3 tubes (Negative control, CMV antigen, and mitogen control). A robust IFN-γ response in the CMV antigen tube indicates immunity to CMV. No correction for the amount of CD4 or CD8 or ratio CD4/CD8 T-cells exists. Patients were considered Quantiferon-CMV positive if they obtained a value above 0.2 IU/mL of INFgamma, as defined by the manufacturer.

#### Autoimmune-induced immune response

Immune response against specific self-antigens was evaluated with anti-nuclear antibody titers (titers ≥ 1:250 were considered as positive) and, in HLA-A*0201 positive patients, with percentages of CD8+ T-cells staining positive with actin-, vimentin-, lamin-loaded HLA-A*0201 tetramers.

#### Microbial translocation

We measured the level of bacterial 16SrDNA as direct marker of microbial translocation together with the level of soluble CD14 (sCD14) as a surrogate marker because LPS measurements in plasma yielded hardly detectable and inconsistent values [24]. Levels of sCD14 were quantified in diluted (1/50) plasma samples tested in duplicates, using an ELISA assay (Diaclone, Connecticut, USA). Plasma 16SrDNA was measured by quantitative polymerase chain reaction (PCR). Degenerate forward and reverse primers, (8F: 5’- AGAGTTTGATYMTGGCTCAG-3’; 361R: 5’-CGYCCATTGBGBAADATTCC-3’), and TaqMan probe (338P:5’-FAM-TACGGGAGGCAGCAGT-BHQ1-3’) for 16SrDNA were synthesized by Eurofins MWG biotech. A standard curve was created from serial dilutions of a plasmid DNA (pCR2.1 TOPO-16SrDNA) containing known copy numbers of the template.

#### Quantification of IFN-α-stimulated-genes: ADAR, ISG15, IFIT1, Mx1 and OAS1 gene expression

IFN-α gene expression was assessed using quantitative reverse transcription polymerase chain reaction (qRT-PCR) of five IFN-α-stimulated-genes: ADAR, ISG15, IFIT1, Mx1 and OAS1 [31]. Total RNA was extracted from peripheral blood mononuclear cells (obtained after Ficoll preparation and storage with Protector RNAse inhibitor (Roche applied biosciences), using the High pure RNA isolation kit (Roche applied biosciences). RNA extraction quality control was measured with the Agilent 2100 bioanalyzer in combination with Agilent RNA 6000 Nano kit (RNA sample quality was considered if the obtained RIN was >7). A total of 300 ng of RNA was reverse transcribed into cDNA using the Transcriptor Reverse Transcriptase kit (Roche applied biosciences). PCR was performed (RT2 SYBR Green qPCR MasterMix, SA Biosciences) in 96-wells plates using LightCycler 480 (Roche Diagnostics) to determine the expression levels of ADAR, ISG15, IFIT1, Mx1 and OAS1 target genes. IFN-α gene expression measurements were expressed as fold change which is calculated as a ratio of averages from control and test sample values.

#### Chronic immune T-cell activation (outcome)

Activation of T cells, particularly of CD8+ T cells, is a hallmark of chronic HIV infection. Levels of CD38+ on CD8+ cells are increased in chronic HIV infection and strongly correlate with plasma viremia [32]. The expression of the CD38+ is found on naive T-cells and as well on activated T-cells, whereas the expression of the HLA-DR molecule on CD8+ T cells strictly reflects the activation of T-cells. Thus, expression of activation markers including CD38 and/or HLA-DR is commonly monitored and assimilated to the degree of systemic immune activation. Of note, in patients with systemic lupus erythematosus, the expression of HLA-DR marker on CD8+ T lymphocytes correlates with disease activity [33]. Our aim was to characterize autoimmune response further in chronic HIV, this is why we chose the co-expression of CD38/HLA-DR on CD8+ T cells as outcome to assess chronic immune T-cell activation. The percentage of HLA-DR+CD38+CD8+T-cells among the CD8+ T-cell subset was evaluated in blood samples by flow cytometry.

### Statistical method and model assignment

#### Structural equation models

Structural equation modelling with latent variables was applied to perform the integrative analysis of available biomarkers in the ACTHIV study. Structural equation models are generalizations of linear regression models, allowing for measurement error in the explanatory as well as the dependent variables. The models consist of analyses that enable direct and indirect effects between variables. The central research question often involves quantities (i.e. “latent variables”), that are unobserved variables for which no direct measurements exist. Existence of these “latent variables” may be revealed by associations among measured variables [34]. Latent variables translate the fact that several observed variables could be imperfect measurements of a single underlying concept [35, 36].

The system of structural equation models is typically decomposed into two submodels: the measurement model and the structural model [29]. Firstly, the measurement model defines the construction of the latent variables with the corresponding observed measured variables (termed “indicators”), assumed to be correlated with the latent variable. In simple words, indicators are quantitative variables assumed to be normally distributed and the model for the latent variables consists of a system of linear regression models of the indicators. In this study, latent variables were built with quantitative biomarkers only. Secondly, the structural model specifies the pattern by which latent variables and observed variables influence each other, either directly or either indirectly.

#### Initially hypothesized model

On the basis of the background literature, we hypothesized direct effects of CMV-induced, autoimmune-induced immune responses and microbial translocation on chronic immune activation and also indirect effects (mediating role) of these factors through IFN-α gene expression. The main integrative analysis was performed in HLA-A*0201 positive patients because biomarkers (tetramers), more specific than anti-nuclear antibody titers in chronic HIV [23] to characterize autoimmune-induced immune response were available in HLA-A*0201 positive patients only.

**Fig 1:**
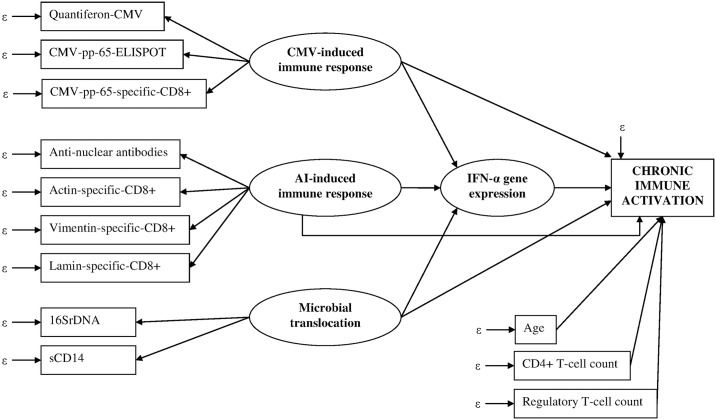
Initially hypothesized model representing the effects of cytomegalovirus-induced, autoimmune-induced immune responses and microbial translocation on chronic immune activation (model adjusted on age, CD4+ T-cell count and regulatory T-cell count). **ACTHIV study**. *Boxes*: *observed variables; ellipses*: *latent variables; ε*: *measurement errors*.

We considered four latent variables supposed to be linked with chronic immune activation:

- CMV-induced immune response latent variable measured by Quantiferon-CMV, CMV-pp65-ELISPOT and CMV-pp-65-specific-CD8+T-cells (as latent variables were built with quantitative biomarkers only, CMV seropositivity was not considered for assessing CMV-induced immune response latent variable),
- autoimmune-induced immune response latent variable measured by anti-nuclear antibody titers and percentages of CD8+ T-cells staining positive with HLA-A0201 tetramers loaded with peptides from actin-, vimentin-, lamin-autoantigens,
- microbial translocation latent variable measured by 16SrDNA and sCD14 in plasma,
- IFN-α latent variable measured by expression levels of 5 IFN-α-stimulated genes. The initial hypothesized model is presented in Fig 1 (hypothetical relationships between variables identified a priori).

### Statistical analyses

Qualitative variables were described as numbers (percentages) and quantitative variables as medians (first quartile; third quartile), using SAS 9.13/SAS 9.3 (SAS Institute, Cary, NC, USA).

#### Data preprocessing

The response variable, i.e. the percentage of HLA-DR+/CD38+ among CD8+T-cells, was log_10_ transformed. IFN-α gene expression measurements were normalized to three reference genes GADPH, RPL13A, HPRT1 (housekeeping genes) using the 2^−ΔΔCT^ method to correct expression data for differences between samples [37].

#### Testing the initial model validity

The goal of structural equation modeling analysis was to determine whether our hypothesized theoretical model (Fig 1), based on relevant literature, was supported by our sample data to reflect this theory. The consistency was evaluated through model-data fit, which indicated the extent to which the postulated network of relations among variables was plausible.

Structural equation models were conducted using data from subjects with no missing data for the variables included in the model (complete case analysis). Structural equation models were performed with R statistical software, using Lava package for linear latent variable models [38]. Parameters were obtained by maximum likelihood estimation. We started the modeling with the initially hypothesized model (Fig 1). Non-significant pathways were dropped to optimize the measurement of latent variables. The simplest and best explanation model for available data (final model) was the one maintaining the hypothesized general pathway identified from goodness-of-fit criteria. Goodness of fit was assessed using three indexes [39, 40]: the chi-square test, the Standardized Root Mean Square Residual (SRMR) and the incremental Bentler’s Comparative Fit Index (CFI). Non-significant p-values at a 0.05 threshold for chi-square test, SRMR values below 0.08 and Bentler’s CFI value above 0.95 indicate a good fit. Structural equation modelling results were reported as standardized estimates βstd and p-values. Standardized estimates represent the expected change in standard deviation units of a variable, due to one standard deviation increase in another. As a unit free representation, they allow comparisons of the relative strength of associations and thus easier interpretation of the coefficients.

#### Sensitivity analyses

We considered the use of the percentage of HLA-DR+T-cells among CD8+ blood T-lymphocytes as the outcome to define chronic immune activation in the HLA-A*0201 positive sample. In addition an analysis in the overall sample was performed considering the CMV-induced immune response measured by Quantiferon-CMV (positive, i.e. ≥ 0.2 IU/mL vs. negative, i.e. < to 0.2 IU/mL) and the autoimmune-induced immune response measured by anti-nuclear antibody titers (positive, i.e. ≥ 1:250 vs. negative, i.e. < 1:250).

## Results

### Patients’ characteristics

A total of 191 patients were enrolled in the ACTHIV study (women: 25%). Median age at inclusion was 50 years old (43; 58). Median duration of viral suppression was 6.1 years (4; 8.5). Median CD4+ and CD8+ T-cell counts were 517 cells/μL (387; 720) and 676 cells/μL (480; 977). Patients had a median CD4+ nadir of 188 cells/μL (89; 275).

Of the 191 patients, 87 were HLA-A*0201 positive patients. There were no differences between HLA-A2 patients and the overall population except for the availability of measurements of percentages of CMV-pp65-specific tetramers CD8+ and actin-, vimentin-, lamin-specific CD8+T-cells in the HLA-A*0201 population (restricted tests).

Demographic, clinical and biological characteristics of the 87 HLA-A*0201 positive patients were representative of the overall population of 191 patients (Table 1A and 1B).

**Table 1:** Demographic, clinical and biological patients’ characteristics. ACTHIV study.

A		
Characteristics	Statistics*	
	All (n = 191)	HLA-A2 (n = 87)
Female	47 (25)	20 (23)
Age at inclusion	50 (43; 58)	50 (44; 60)
Risk factors		
• Homo-bisexual—MSM	92 (48)	43 (49)
• Injection drug users/Blood products	1/1 (0.5/0.5)	1/0 (1.5/0)
• Heterosexual	81 (42)	34 (39)
• Other risk factors	16 (8)	9 (10.5)
CDC stage A	101 (53)	46)
CDC Stage B	48 (25)	33)
CDC Stage C	42 (22)	18 (21)
Duration since first reported seropositivity, years	12.3 (8; 18)	12.5 (7.5; 19)
Duration of viral suppression, years	6.1 (4; 8.5)	5.8 (4.4; 7.7)
Duration of ART exposure, years	9.7 (6; 13)	10.5 (5.9; 13.4)
cART at baseline		
• 2NRTI+1NNRTI	65 (34)	24 (28)
• 2NRTI+1PI	100 (52)	48 (55)
• Others	26 (14)	15 (17)
***CD4+ cells***		
CD4+ nadir, cells/μL	188 (89; 275)	172 (47; 249)
CD4+, cells/μL	517 (387; 720)	441 (328; 660)
CD4+, %	42 (33; 50)	42 (33; 48)
% HLA-DR+/CD38+ among CD4+ T-cells	4.5 (3.0; 6.5)	4.6 (3.0; 6.3)
**B**		
***CD8+ cells***		
CD8+, cells/μL	676 (480; 977)	652 (478; 896)
% CD8+	53 (46; 62)	54 (48; 62)
% HLA-DR+/CD38+ among CD8+ T-cells	16.8 (11.1; 23.2)	15.8 (11.1; 22.6)
**Regulatory T-cells**, cells/μL	38 (25; 51)	35 (22; 49)
**B lymphocytes**, cells/μL	197 (137; 309)	197 (129; 274)
**Natural killer lymphocytes**, cells/μL	80 (30; 168)	81 (27; 192)
**Cytomegalovirus-related variables**		
CMV Serology positive	177 (93)	79 (91)
CMV PCR positive (whole blood)	1 (0.5)	0 (0)
Quantiferon-CMV positive	148 (78)	71 (82)
Quantiferon-CMV values, IU/mL	3.3 (0.3; 9.8)	5.3 (0.9; 9.9)
CMV-pp65-ELISPOT†	377.5 (120.0; 689.0)	452 (120; 728)
% CMV-pp-65-specific-CD8+T-cells	NA¶	1.1 (0.1; 2.1)‡
**Autoimmune response related variables**		
Antinuclear antibody positive	43 (26)‡	21 (28)‡
% Actin-specific-CD8+ T-cells	NA	0.03 (0.02; 0.06)‡
% Vimentin-specific-CD8+ T-cells	NA	0.01 (0.01; 0.02)‡
% Lamin-specific-CD8+ T-cells	NA	0.01 (0.01; 0.02)‡
**Microbial translocation related variables**		
sCD14 μg/mL	1.9 (1.6; 2.2)	1.8 (1.6; 1.9)
16S rDNA log_10_ copies/mL	3.2 (3.0; 3.5)	3.3 (3.1; 3.4)

* for categorical variables n (%), for quantitative variables median (first quartile; third quartile) are presented. NRTI, nucleoside reverse-transcriptase inhibitor; NNRTI, non-nucleoside reverse-transcriptase inhibitor; PI, protease inhibitor; MSM, men who have sex with men; CDC, Centers for Disease Control and Prevention; cART, antiretroviral therapy combination.

^†^ Median spot-forming cells / 2.5×105 peripheral blood mononuclear cells.

^‡^ Less than 10% of missing values.

^¶^ Not applicable.

### Measurement of the processes

Main patient’s characteristics of variables related to CMV-, autoimmune-induced immune responses and microbial translocation are reported in Table 1B. In the overall population, Quantiferon-CMV was positive in 78% of patients and antinuclear antibodies were positive in 26% of patients. In the HLA-A*0201 positive patients, Quantiferon-CMV was positive in 82% and antinuclear antibodies were positive in 28% of patients. Levels of the tested self-antigen-specific T cells were low in this study. Levels of 16SrDNA and sCD14 for studying microbial translocation did not differ between the HLA-A*0201 positive patients and the overall population (Table 1B).

**Fig 2:**
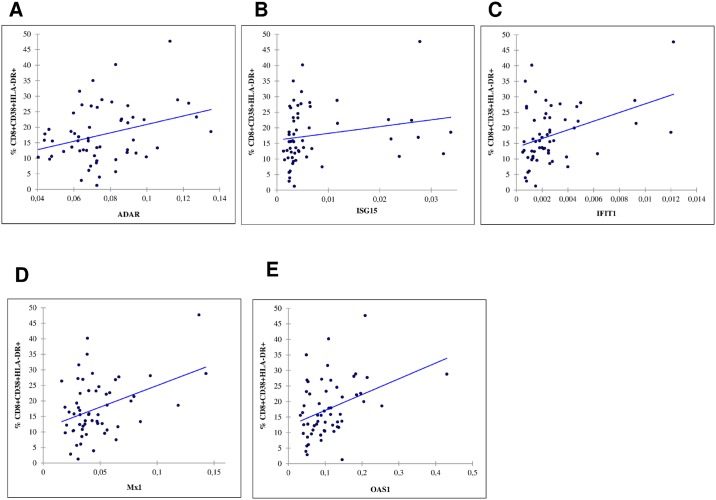
Spearman correlations between percentages of CD8+CD38+HLA-DR+ T-cells and IFN-α gene expression measurements, HLA-A*0201 positive patients (n = 57). **ACTHIV study**. *A) ADAR*: *r = 0*.*31*, *p-value = 0*.*0180; B) ISG15*: *r = 0*.*34*, *p-value = 0*.*0085; C) IFIT1*: *r = 0*.*32*, *p-value = 0*.*0166; D) Mx1*: *r = 0*.*29*, *p-value = 0*.*0301; E) OAS1*: *r = 0*.*35*, *p-value = 0*.*0077*.

Of the 87 HLA-A*0201 positive patients, the main complete analysis was performed in the 57 patients in whom the percentages of actin-, vimentin-, lamin-specific CD8+T-cells as well as Quantiferon, pp-65 ELISPOT for CMV were available (subjects with missing values for variables included in the model were excluded). The 30 other patients did not differ from the 57 HLA-A*0201 positive patients included in the final analysis. In the 57 patients, median % of HLA-DR+/CD38+CD8+T-cells was 15.8 (11.7; 23.2). Median % actin-specific-CD8+ T cells, % vimentin-specific-CD8+ T cells, % lamin-specific-CD8+ T cells were respectively 0.03 (0.02; 0.06), 0.01 (0.01; 0.02) and 0.01 (0.01; 0.02). Median Quantiferon-CMV values were 5.3 IU/mL (0.4; 11.5) and median CMV-pp65-ELISPOT values were 309 (95; 645). IFN-α gene expression measurements were positively and significantly correlated with the percentages of HLA-DR+/CD38+CD8+T-cells (Fig 2).

### Main integrative analysis in HLA-A*0201 positive patients

#### Model building

The model building started with the model represented in Fig 1. As biomarkers of microbial translocation were not associated with chronic immune activation in univariable analysis (16SrDNA effect: p-value = 0.6100; sCD4 effect: p-value = 0.9750; microbial translocation latent variable: p-value = 0.9922) and in multivariable analysis performed in our previous work [24], they were dropped from the final model. Anti-nuclear antibodies were not contributive for the definition of the autoimmune-induced immune response latent variable (p-value = 0.2785) as well as CMV-pp65-specific-CD8+ T-cells for the definition of the CMV-induced immune response latent variable (p-value = 0.7049). Therefore, further analyses included the effect of CMV-induced immune response measured by Quantiferon and pp-65 ELISPOT, autoimmune-induced immune response measured by actin, vimentin, lamin-specific CD8+, interferon-α pathway measured by the expression level of the five interferon signaling genes (ADAR, ISG15, IFIT1, Mx1 and OAS1) and chronic immune activation.

#### Final model

**Fig 3:**
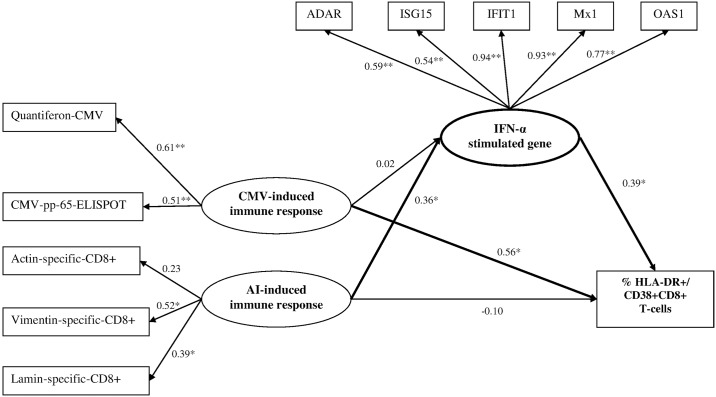
Final hypothesized model representing the effects of cytomegalovirus-induced-, autoimmune-induced immune responses latent variables on chronic immune activation. **Figure to read in conjunction with** Table 2. **ACTHIV study**. *Boxes*: *observed variables; ellipses*: *latent variables; measurement errors deleted for clarity*.*p-values < 0.*05*. **p-values < 0.*0001*.

The final best-fit model for ACTHIV data is shown in Fig 3 ant the corresponding results are presented in Table 2.

**Table 2:** Effects of cytomegalovirus-, autoimmune-induced immune responses on chronic immune activation (HLA-DR+/CD38+CD8+ T-cells log10%), HLA-A*0201 positive patients (n = 57). Main integrative analysis to read in conjunction with Fig 3. ACTHIV study.

Process	βstd*	p value
**Measurement models (latent variable definitions)**		
**Cytomegalovirus(CMV)-induced immune response latent variable**		
CMV response latent variable → Quantiferon-CMV	0.61	< 0.0001
CMV response latent variable → CMV-pp-65-ELISPOT	0.51	< 0.0001
**Autoimmune(AI)-induced immune response latent variable**		
AI response latent variable → % actin-specific-CD8+	0.23	0.0862
AI response latent variable → % vimentin-specific-CD8+	0.52	0.0006
AI response latent variable → % lamin-specific-CD8+	0.39	0.0184
**IFN-α-stimulated-genes latent variable**		
IFN-α-stimulated-genes latent variable → ADAR	0.59	< 0.0001
IFN-α-stimulated-genes latent variable → ISG15	0.54	< 0.0001
IFN-α-stimulated-genes latent variable → IFIT1	0.94	< 0.0001
IFN-α-stimulated-genes latent variable → Mx1	0.93	< 0.0001
IFN-α-stimulated-genes latent variable → OAS1	0.77	< 0.0001
**Structural model**		
CMV response latent variable → IFN-α-stimulated-genes latent variable	0.02	0.9069
CMV response latent variable → % HLA-DR+/CD38+CD8+	0.56	**0.0004**
AI response latent variable → IFN-α-stimulated-genes latent variable	0.36	**0.0401**
AI response latent variable → % HLA-DR+/CD38+CD8+	-0.10	0.3169
IFN-α-stimulated-genes latent variable → % HLA-DR+/CD38+CD8+	0.39	**0.0044**

* Standardized estimates

Quantiferon-CMV (βstd = 0.61) and CMV-pp-65-ELISPOT (βstd = 0.51) contributed significantly to the CMV-induced response. The positive significant standardized estimates mean that higher levels of CMV-induced response were associated with higher levels of Quantiferon-CMV and CMV-pp-65-ELISPOT. Autoimmune-induced response was composed by % actin-specific-CD8+ T cells, % vimentin-specific-CD8+ T cells and % lamin-specific-CD8+ T cells. All standardized estimates were positive. The percentage of vimentin-specific-CD8+ T cells presented the highest contribution to explain the autoimmune-induced response variation (βstd = 0.52, p-value = 0.0006). IFN-α gene latent variable was composed of expression levels of IFN-α-stimulated genes: ADAR (βstd = 0.59), ISG15 (βstd = 0.54), IFIT1 (βstd = 0.94), Mx1 (βstd = 0.93), OAS1 (βstd = 0.77).

The analysis of the effect of each single process revealed a direct association of chronic immune activation of CD8+ T-cells with CMV-induced response (βstd = 0.42, p-value = 0.0232), and with IFN-α genes (βstd = 0.31, p-value = 0.0093). No direct effect of autoimmune-induced response and chronic immune activation was found (βstd = 0.04, p-value = 0.7361). However, autoimmune-induced response was associated with IFN-α genes (βstd = 0.33, p-value = 0.0457).

The structural model including all processes presented in Fig 3 revealed a direct positive significant effect of the CMV-induced response on chronic immune activation (βstd = 0.56, p-value = 0.0004) but no significant association with the IFN-α genes (p-value = 0.9069). There was no direct significant association of autoimmune-induced response and chronic immune activation (p-value = 0.3169). The effect of autoimmune-induced response on chronic immune activation was indirect through IFN-α genes: autoimmune-induced response was significantly associated with IFN-α genes (βstd = 0.36, p-value = 0.0401) and IFN-α genes were associated with chronic immune activation (βstd = 0.39, p-value = 0.0044). The goodness of fit of the model was fair (chi-square p-value = 0.32; SRMR = 0.07; CFI = 0.98). These findings persisted after adjustment for age, CD4+ T-cell count and regulatory T-cell count (S2 Table).

When defining CD8+ T-cell activation with % CD8+HLA-DR+ T cells (log_10_ transformed) in place of % CD8+CD38+HLA-DR+ as the outcome, the adjusted results were consistent (S3 Table). There was a direct positive significant effect of CMV-induced response on chronic immune activation (βstd = 0.45, p-value = 0.0056) as well as an indirect effect of autoimmune-induced response on chronic immune activation through IFN-α genes. Autoimmune-induced response was significantly associated with IFN-α genes (βstd = 0.35, p-value = 0.0345) and IFN-α genes were associated with chronic immune activation (βstd = 0.35, p-value = 0.0111).

#### Sensitivity analysis in the overall population

Of the 191 patients included in the ACTHIV study, the sensitivity analysis was performed in 142 patients in whom Quantiferon-CMV positive versus negative, anti-nuclear antibody titers positive versus negative, interferon-stimulated genes and % HLA-DR+/CD38+CD8+ T-cells were available (including the 57 patients from the main integrative analysis). In these patients, median % of HLA-DR+/CD38+CD8+T-cells was 16.9 (10.8; 23.7), Quantiferon-CMV was positive in 78% of patients and antinuclear antibodies were positive in 23% of patients. The adjusted results were roughly consistent with previous results (S4 Table). Quantiferon-CMV positivity was associated with higher percentage of CD8+CD38+HLA-DR+ (βstd = 0.15, p-value = 0.0648), positive anti-nuclear antibody titers were associated with higher expression of IFN-α genes (βstd = 0.16, p-value = 0.0797) and higher IFN-α-gene expression was associated with chronic immune activation (βstd = 0.22, p-value = 0.0120).

## Discussion

Exploring the mechanisms driving chronic immune activation in successfully treated HIV-infected patients requires a statistical modelling approach. Testable pathways need to be identified a priori and hypotheses regarding the relationships between multifactorial triggers involved in chronic HIV need to be challenged by this approach. Common strategies for multiple markers are to analyze each marker separately or to include original markers simultaneously as predictors in multivariable regression models, assuming the presence of one single relationship between variables. In addition to neglecting any a priori knowledge, these strategies could lead to invalidate results due to: i) type I error inflation from multiple testings, ii) type II errors (missing true associations) because immune markers only reflect a small part of the variation of the underlying immunological mechanism, iii) estimation problems due to multicollinearity or neglected measurement errors. Thus, to unravel the complexity of research questions addressed by modern immunological studies, sophisticated statistical approaches are necessary [41–44].

In this study, we considered the potential for using SEM to investigate the mediating role of INF-α in chronic immune activation in successfully treated HIV-infected patients. The advantage of SEM is to use all available information in an integrative analysis, avoiding the methodological issues mentioned above. Moreover, latent variables are defined by several markers that could reflect an underlying immunological mechanism and SEM considers a priori knowledge limiting the number of analyses to run and multiple testing. Due to availability of percentages of actin-, vimentin-, lamin-specific CD8+T-cells (HLA-A2-restricted tests) to further characterize autoimmune response, we performed our main analysis in a subsample of HLA-A2 patients making the whole study original compared to the previously published [24]. The mediating role of INF-α has been investigated and underlying activation processes were defined as latent variables, i.e. CMV-induced-, autoimmune-induced immune responses, microbial translocation and INF-α. The best explanation model for the available data was identified from goodness-of-fit-criteria.

SEM final results revealed a direct positive significant effect of CMV-induced response and IFN-α genes on chronic immune activation. In addition, the approach might suggest an indirect effect of autoimmune-induced response on chronic immune activation through IFN-α genes. These findings were consistent in various sensitivity analyses performed in HLA-A*0201 positive patients and in the overall population. Results in the overall population could be explained by the fact that Quantiferon-CMV positivity and positivity of anti-nuclear antibody titers may not be accurate enough for reflecting respectively the CMV-induced response and the autoimmune-induced response leading to a dilution bias and underlying the need for an integrative analysis using all available information.

Particularly, all analyses highlighted the significant effect of IFN-α genes on chronic immune activation, accounting for the evidence of the involvement of the IFN-α pathway reported in chronic immune activation in successfully treated HIV-1-infected patients [10–12].

We confirmed effect of the CMV-induced response on chronic immune activation [24]. Our findings are in agreement with former studies highlighting the involvement of CMV in immunosenescence and chronic HIV infection [45–49]. According to this new analysis, the effect of CMV-induced response on CD8+ T-cell activation was not mediated by the IFN-α- genes measured in the present study (ADAR, ISG15, IFIT1, Mx1 and OAS1). The direct effect of CMV-induced response on chronic immune activation was revealed through the measurements of Quantiferon and pp-65 ELISPOT. CMV-tetramer-pp65-specific-CD8+ T-cells were not contributive to define the CMV-induced immune response latent variable (p = 0.7049). Stone et al [50] reported that percentages of CMV-pp65-specific-CD8+ T-cells were similar in HIV patients with stable undetectable HIV viremia due to highly active antiretroviral therapy and healthy controls. Taking together, these findings indicate that CMV-pp65-specific-CD8+ T-cells in circulating blood may not reflect accurately the status of the response against CMV and thus, question the relevance of measuring them at distance of the primo-infection or of reactivations in blood. Indeed, anti-CMV response may primarily rely on in situ tissue resident memory T-cells [51] which do not recirculate in blood but rather stay in barrier tissue such as lung, skin or intestine. Therefore the lack of correlation between tetramer positive T-cells and CMV does not mean they do not exist but that they are not circulating in the blood we were looking at.

Interestingly, our results might suggest an indirect effect of autoimmune-induced response on chronic immune activation mediated through IFN-α genes. Even in the absence of clinical events, low level biological signals may have consequences such as T-cell activation. The effect of autoimmune-induced immune response on IFN-α latent variable was revealed through the measurements of actin-, vimentin-, lamin-specific CD8+T-cells illustrating the important role of caspase-cleaved apoptotic self-antigens in immune activation during chronic HIV infection, as suggested by Rawson et al [23]. Of note, the percentage of vimentin-specific CD8+T-cells contributed the most to define the autoimmune-induced response latent variable. Moreover, the use of a latent variable seemed particularly relevant regarding the low levels of self-antigen-specific T cells in this study. Indeed, anti-nuclear antibodies did not contribute to the definition of the autoimmune-induced response latent variable and were dropped from the final model. The latter result reveals the need to measure biomarkers more relevant than anti-nuclear antibodies when studying autoimmune-induced response in chronic HIV [23]. Altogether, these findings might provide valuable insights in hypotheses regarding the role of autoimmunity, as there were very few reports in the literature studying auto-immunity in HIV infected patients [22]. However, these findings need to be interpreted carefully because i) there was no direct significant association of autoimmune-induced response with chronic immune activation, ii) even though the use of a latent variable seemed particularly relevant for defining the autoimmune-induced immune response, the very low levels of self-antigen-specific T cells in this study may raise concerns about the existence of a biological autoimmunity affecting immune activation sufficiently.

Previous studies have demonstrated that bacterial translocation (measured by sCD14 and 16SrDNA) was associated with immune activation. In this work, biomarkers of microbial translocation were not associated with chronic immune activation in univariable analysis and in multivariable analysis performed in our previous work [24]. This could be explained by the very specific characteristics of our population: median duration of viral suppression 5.8 years (IQR: 4.4; 7.7), median duration of ART exposure 10.5 years (5.9; 13.4), with very low levels of microbial translocation: median sCD14 μg/mL 1.8 (1.6; 1.9), median 16S rDNA log10 copies/mL 3.3 (3.1; 3.4) (data for HLA-A2 patients). Moreover, Abad-Fernandez M et al did not find any correlation between sCD14 or bacterial rDNA with activated CD8 T cells in long-term suppressed HIV-1-infected individuals [52].

Our study also had some limitations. First, the ACTHIV study is a cross-sectional study nested in the ANRS CO3 Aquitaine Cohort not allowing to infer the temporality and causal direction of the observed associations. Enrolled patients initiated antiretroviral therapy between 2005 and 2008 and were virologically suppressed for at least two years. Median duration of viral suppression was 6.1 years (4; 8.5) in the overall population and 5.8 years (4.4; 7.7) in the HLA-A2 population. We had no other data on persistent ongoing HIV replication. This is a clear limitation of the study. Second, even though our results were confirmed in all sensitivity analyses, the main complete case integrative analysis was performed in 66% (57/87) HLA-A*0201 positive patients, accounting for a potential lack of power that may have affected results on the effects of autoimmunity and SEM model identifiability (generally the greater the model complexity, the more observations are needed), questioning the need for additional methods such as multiple imputation. Third, we had only five quantitative gene expression measurements of IFN-α-stimulated-genes. Some studies report more specific pathways of IFN-α that could be involved in HIV chronic immune activation, i.e. IFN-α MYD88 dependent pathway and IFN-α MYD88 independent pathway [53–55]. Additional data on these pathways may have helped to better characterize the potential indirect effects of CMV or microbial translocation on chronic immune activation. Further research is needed and could be implemented in a longitudinal framework, allowing the inference of causal relationships. Combining appropriate biomarkers and adequate quantitative gene expression measurements, including IFN-α, using structural equation modelling could also be applied in longitudinal data analysis [56]. Indeed, ongoing efforts are made to identify and better characterize the IFN-α-stimulated-genes landscape [57, 58].

We focused on chronic immune T-cell activation, proven to be a leading factor for HIV disease progression but we acknowledge the importance of other markers including B cells, Natural Killer cells, dendritic cells, monocytes, macrophages and neutrophils of the inflammation cascade, as well as of the endothelium and the coagulation system in immune activation [59]. We do feel that this study additionally illustrates the potential of latent variables, in modern immunology, to assess biological processes that cannot be measured directly.

In this study, we used structural equation modeling analysis to determine whether our hypothesized theoretical model (Fig 1) was supported by sample data to reflect our theory in chronic HIV. The simplest and best explanation model for available data maintaining the hypothesized general pathway (Fig 3) passed the goodness-of-fit tests. As estimation techniques, modeling capacities and breadth of SEM applications are expanding rapidly, beyond social sciences, to embrace multiple fields as modern immunology [41, 43, 44], endocrinology [60], cancer [61, 62] or environment [63], we would like to warn readers about SEM results that have to be interpreted carefully. Indeed, because a model fits the data well does not mean that the model reflects perfectly the reality. Other statistical plausible models could have fitted the data [29] but the advantage of SEM is to consider prior knowledge limiting the number of analyses to run and multiple testing. This is why, beyond the need to report a detailed statistical justification of the model (specification, identifiability, estimation, goodness of fit) [29], we highlight the importance to integrate the expertise of specialists in the field when defining the testable pathways and when designing the initial hypothesized theoretical model. Particularly, carefully conducted SEM analyses with a minimal number of weak assumptions provide an important supplement to standard regression results (i.e. mediation, measurement error in explanatory variables). In conclusion, this study not only provides valuable insights into underlying mechanisms of chronic immune activation in HIV-infection but also illustrates the potential of SEM for handling multiple relationships, understanding epidemiological processes using observational data. Furthermore, the results clearly highlight the need for integrative analyses to make the most out of immunological data [64, 65]. Using this flexible framework, the role of CMV-immune response on the CD8+ T cell activation has been confirmed, encouraging intervention against CMV in successfully treated HIV-1-infected patients [21, 66]. Our results further add to the growing body of evidence that IFN-α is involved in chronic immune activation. The signal found for the potential role of auto-immunity response in chronic immune activation requires further investigations.

## Supporting Information

S1 Table Univariable and multivariable linear regressions of HLA-DR+/CD38+CD8+ T-cells (log10%). ACTHIV study.
(DOC)

S2 Table Effects of cytomegalovirus-, autoimmune-induced immune responses on chronic immune activation (HLA-DR+/CD38+CD8+ T-cells log10%), adjusted for age, CD4+ T-cell count and regulatory T-cell count. HLA-A*0201 positive patients (n = 57), ACTHIV study.
(DOCX)

S3 Table Effects of cytomegalovirus-, autoimmune-induced immune responses on chronic immune activation (% HLA-DR+/CD8+), adjusted for age, CD4+ T-cell count and regulatory T-cell count. HLA-A*0201 positive patients (n = 57), ACTHIV study.
(DOCX)

S4 Table Effects of Quantiferon-CMV positivity & positive anti-nuclear antibody titers on chronic immune activation (% HLA-DR+/CD38+CD8+), adjusted for age, CD4+ T-cell count and regulatory T-cell count (n = 142), ACTHIV study. (DOCX)
