# Supplementary material for: Integrative Analysis of Immunological Data to Explore Chronic Immune T-Cell Activation in Successfully Treated HIV Patients
Source: PLoS One. 2017 Jan 3;12(1):e0169164. doi: 10.1371/journal.pone.0169164 (PMC5207686; doi:10.1371/journal.pone.0169164)
Supplement: S1 Table — ACTHIV study. (DOC) [file pone.0169164.s001.doc]

**S1 Table. Univariable and multivariable linear regressions of HLA-DR+/CD38+CD8+ T-cells (log_10_ %). ACTHIV study.**

|  | **Univariable** | | | |  |  | **Multivariable (n=167) R²=0.16** | | | |  | **Multivariable (n=165) R²=0.14** | | | |
| --- | --- | --- | --- | --- | --- | --- | --- | --- | --- | --- | --- | --- | --- | --- | --- |
| **Variables** | **Reg**  **coeff** | **Std** | **Stand**  **coeff** | ***P*** | **R²** |  | **Reg**  **coeff** | **Std** | **Stand**  **coeff** | ***P*** |  | **Reg**  **coeff** | **Std** | **Stand**  **coeff** | ***P*** |
| CMV serology pos vs neg | 0.226 | 0.070 | 0.23 | 0.0014 | 0.05 |  | 0.215 | 0.077 | 0.2 | 0.006 |  | **-** | **-** | **-** | - |
| Quantiferon-CMV pos vs neg | 0.130 | 0.045 | 0.21 | 0.0042 | 0.04 |  | - | - | - | - |  | 0.106 | 0.047 | 0.17 | 0.0238 |
| CMV-pp65-ELISPOIT pos vs neg | 0.191 | 0.062 | 0.22 | 0.0025 | 0.05 |  | - | - | - | - |  | - | - | - | - |
| CMV-pp65-specific-CD8+ pos vs neg | 0.144 | 0.079 | 0.20 | 0.0716 | 0.04 |  | - | - | - | - |  | - | - | - | - |
| Actin-specific CD8+ T-cells pos vs neg | 0.098 | 0.064 | 0.18 | 0.1290 | 0.03 |  | - | - | - | - |  | - | - | - | - |
| Age for one year higher | 0.005 | 0.002 | 0.22 | 0.0026 | 0.05 |  | 0.004 | 0.002 | 0.18 | 0.0161 |  | 0.004 | 0.002 | 0.18 | 0.0194 |
| CD4+ T-cells for 100 cells/µL higher | -0.023 | 0.006 | -0.26 | 0.0003 | 0.07 |  | -0.015 | 0.009 | -0.17 | 0.0929 |  | -0.015 | 0.009 | -0.17 | 0.1144 |
| 16SrDNA for one log_10_ cp/mL higher | -0.081 | 0.055 | -0.11 | 0.1431 | 0.01 |  | -0.048 | 0.052 | -0.07 | 0.3612 |  | -0.080 | 0.053 | -0.11 | 0.1366 |
| Regulatory T-cells for one cell/µL higher | -2.595 | 0.793 | -0.23 | 0.0013 | 0.05 |  | -0.627 | 1.151 | -0.06 | 0.5867 |  | -0.742 | 1.166 | -0.07 | 0.5255 |
|  | | | | | | | | | | | | | | | |
|  | **Multivariable (n=164) R²=0.14** | | | |  |  | **Multivariable (n=70) R²=0.20** | | | |  | **Multivariable (n=58) R²=0.17** | | | |
| **Variables** | **Reg**  **coeff** | **Std** | **Stand**  **coeff** | ***P*** |  |  | **Reg**  **coeff** | **Std** | **Stand**  **coeff** | ***P*** |  | **Reg**  **coeff** | **Std** | **Stand**  **coeff** | ***P*** |
| CMV serology pos vs neg | - | - | - | - |  |  | **-** | **-** | **-** | - |  | **-** | **-** | **-** | - |
| Quantiferon-CMV pos vs neg | - | - | - | - |  |  | - | - | - | - |  | - | - | - | - |
| CMV-pp65-ELISPOT pos vs neg | 0.166 | 0.067 | 0.18 | 0.0141 |  |  | - | - | - | - |  | - | - | - | - |
| CMV-pp65-specific-CD8+ pos vs neg | - | - | - | - |  |  | 0.176 | 0.09 | 0.23 | 0.0538 |  | - | - | - | - |
| Actin-specific CD8+ T-cells pos vs neg | - | - | - | - |  |  | - | - | - | - |  | -0.178 | 0.075 | -0.03 | 0.8124 |
| Age for one year higher | 0.004 | 0.002 | 0.18 | 0.0204 |  |  | 0.007 | 0.002 | 0.28 | 0.0225 |  | 0.008 | 0.003 | 0.34 | 0.0170 |
| CD4+ T-cells for 100 cells/µL higher | -0.016 | 0.009 | -0.18 | 0.0900 |  |  | -0.020 | 0.015 | -0.24 | 0.1729 |  | -0.027 | 0.016 | -0.34 | 0.0976 |
| 16SrDNA for one log_10_ cp/mL higher | -0.058 | 0.053 | -0.08 | 0.2698 |  |  | -0.115 | 0.087 | -0.16 | 0.1898 |  | -0.094 | 0.088 | -0.14 | 0.2925 |
| Regulatory T-cells for one cell/µL higher | -0.550 | 1.164 | -0.05 | 0.6374 |  |  | 0.830 | 1.919 | 0.08 | 0.6668 |  | 1.850 | 2.180 | 0.17 | 0.4001 |

Legend: Std: Standard deviation; Reg coeff, regression coefficient; Stand coeff, Standardized regression coefficient. The regression coefficient indicates the difference in HLA-DR+/CD38+CD8+ T-cells in log_10_% for one unit of the adjustment variables. For example, looking at results of the model including the variable Quantiferon-CMV: In comparison with a patient of same age, with the same 16SrDNA values and the same regulatory T-cell count a patient with 100 CD4+ cells/μl more has 0.015 log_10_% HLA-DR+/CD38+CD8+ T-cells less. The standardized coefficients are computed by multiplying the regression coefficient by the sample standard deviation of the variable and by dividing it by the standard deviation of the outcome variable. The standardized coefficient is interpretable as the average change in the outcome variable, in standard-deviation units, for a one standard-deviation increase in an explanatory variable, holding constant the other explanatory variables. If there are just two variables, the outcome variable and one explanatory variable, then the standardized coefficient equals the correlation coefficient. Thus, standardized regression coefficients allow seeing the relative importance of each of the explanatory variable included in the model to explain the outcome variable.
