# Supplementary material for: Integrative Analysis of Immunological Data to Explore Chronic Immune T-Cell Activation in Successfully Treated HIV Patients
Source: PLoS One. 2017 Jan 3;12(1):e0169164. doi: 10.1371/journal.pone.0169164 (PMC5207686; doi:10.1371/journal.pone.0169164)
Supplement: S3 Table — HLA-A*0201 positive patients (n = 57), ACTHIV study. (DOCX) [file pone.0169164.s003.docx]

**S3 Table. Effects of cytomegalovirus-, autoimmune-induced immune responses on chronic immune activation (% HLA-DR+/CD8+), adjusted for age, CD4+ T-cell count and regulatory T-cell count. HLA-A*0201 positive patients (n=57), ACTHIV study.**

| **Process** | **βstd*** | **p value** |
| --- | --- | --- |
| **Measurement models (latent variable definitions)**  **Cytomegalovirus(CMV)-induced immune response latent variable**  CMV response latent variable 🡪 Quantiferon-CMV  CMV response latent variable 🡪 CMV-pp-65-ELISPOT  **Autoimmune(AI)-induced immune response latent variable**  AI response latent variable 🡪 % actin-specific-CD8+  AI response latent variable 🡪 % vimentin-specific-CD8+  AI response latent variable 🡪 % lamin-specific-CD8+  **IFN-α-stimulated-genes latent variable**  IFN-α-stimulated-genes latent variable 🡪 ADAR  IFN-α-stimulated-genes latent variable 🡪 ISG15  IFN-α-stimulated-genes latent variable 🡪 IFIT1  IFN-α-stimulated-genes latent variable 🡪 Mx1  IFN-α-stimulated-genes latent variable 🡪 OAS1  **Structural model**  CMV response latent variable 🡪 IFN-α-stimulated-genes latent variable  CMV response latent variable 🡪 % HLA-DR+/CD8+  AI response latent variable 🡪 IFN-α-stimulated-genes latent variable  AI response latent variable 🡪 % HLA-DR+/CD8+ | 0.51  0.62  0.23  0.52  0.39  0.58  0.54  0.94  0.92  0.76  -0.05  0.45  0.35  -0.16 | < 0.0010  < 0.0001  0.0800  0.0003  0.0158  < 0.0001  < 0.0001  < 0.0001  < 0.0001  < 0.0001  0.7656  **0.0056**  **0.0345**  0.1722 |
| IFN-α-stimulated-genes latent variable 🡪 % HLA-DR+/CD8+ | 0.35 | **0.0111** |
| Age 🡪 % HLA-DR+/CD8+ | 0.22 | 0.0542 |
| CD4+ T-cell count 🡪 % HLA-DR+/CD8+ | -0.21 | 0.2089 |
| Regulatory T-cell count 🡪 % HLA-DR+/CD8+ | 0.10 | 0.5521 |

Legend: *Standardized estimates
