# Supplementary material for: Integrative Analysis of Immunological Data to Explore Chronic Immune T-Cell Activation in Successfully Treated HIV Patients
Source: PLoS One. 2017 Jan 3;12(1):e0169164. doi: 10.1371/journal.pone.0169164 (PMC5207686; doi:10.1371/journal.pone.0169164)
Supplement: S4 Table — (DOCX) [file pone.0169164.s004.docx]

**S4 Table. Effects of Quantiferon-CMV positivity & positive anti-nuclear antibody titers** **on chronic immune activation (% HLA-DR+/CD38+CD8+), adjusted for age, CD4+ T-cell count and regulatory T-cell count (n=142), ACTHIV study.**

| **Process** | **βstd*** | **p value** |
| --- | --- | --- |
| **Measurement models (latent variable definitions)**  **IFN-α-stimulated-genes latent variable**  IFN-α-stimulated-genes latent variable 🡪 ADAR  IFN-α-stimulated-genes latent variable 🡪 ISG15  IFN-α-stimulated-genes latent variable 🡪 IFIT1  IFN-α-stimulated-genes latent variable 🡪 Mx1  IFN-α-stimulated-genes latent variable 🡪 OAS1  **Structural model**  Quantiferon-CMV pos vs neg 🡪 IFN-α-stimulated-genes latent variable  Quantiferon-CMV pos vs neg 🡪 % HLA-DR+/CD38+CD8+  Anti-nuclear antibody titers pos vs neg 🡪 IFN-α-stimulated-genes latent variable  Anti-nuclear antibody titers pos vs neg 🡪 % HLA-DR+/CD38+CD8+ | 0.64  0.47  0.52  0.90  0.78  0.06  0.15  0.16  0.04 | < 0.0001  < 0.0001  < 0.0001  < 0.0001  < 0.0001  0.5175  **0.0648**  **0.0797**  0.6168 |
| IFN-α-stimulated-genes latent variable 🡪 % HLA-DR+/CD38+CD8+ | 0.22 | **0.0120** |
| Age 🡪 % CD8+CD38+HLA-DR+ | 0.18 | 0.0253 |
| CD4+ T-cell count 🡪 % HLA-DR+/CD38+CD8+ | -0.18 | 0.0925 |
| Regulatory T-cell count 🡪 % HLA-DR+/CD38+CD8+ | 0.01 | 0.9951 |

Legend: *Standardized estimates
